# Effect of COVID-19 mRNA Vaccine on Human Lung Carcinoma Cells *In Vitro* by Means of Raman Spectroscopy and Imaging

**DOI:** 10.1021/acsomega.3c05287

**Published:** 2023-10-30

**Authors:** Halina Abramczyk, Jakub Surmacki

**Affiliations:** Department of Chemistry, Institute of Applied Radiation Chemistry, Laboratory of Laser Molecular Spectroscopy, Lodz University of Technology, Wróblewskiego 15, 93-590 Łódź, Poland

## Abstract

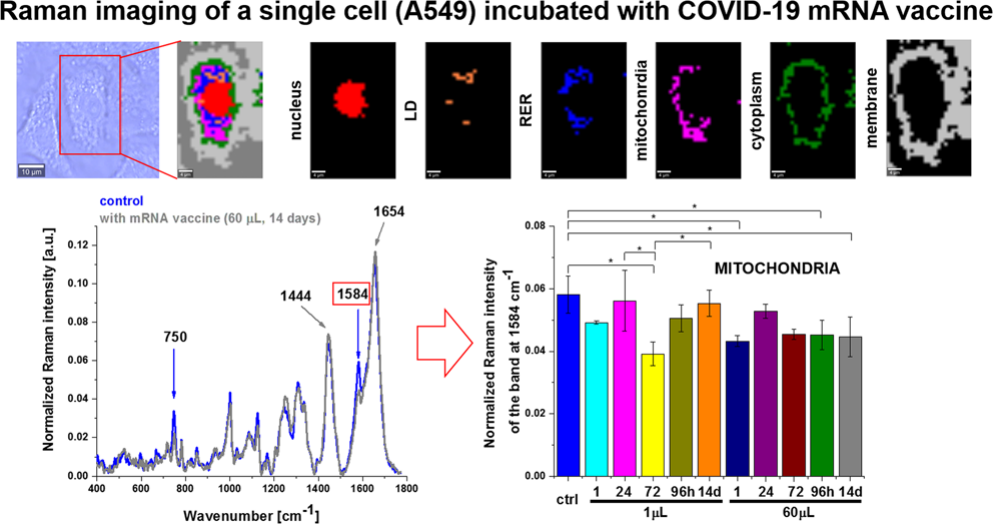

The effect of COVID-19 mRNA vaccine on human lung epithelial carcinoma cells (A549) *in vitro* as a convenient preclinical model was studied by means of Raman spectroscopy and imaging. The article focuses on Raman imaging as a tool to explore apoptosis and oxidative phosphorylation in mitochondrial dysfunctions. The Raman results demonstrate alterations in the oxidation–reduction pathways associated with cytochrome *c*. We found that the COVID-19 mRNA vaccine downregulates the concentration of cytochrome *c* upon incubation with tumorous lung cells. The concentration of the oxidized form of cytochrome *c* in the mitochondria of lung cells decreases upon incubation with the COVID-19 mRNA vaccine. A lower concentration of oxidized cytochrome *c* in mitochondria illustrates lower effectiveness of oxidative phosphorylation (respiration), reduced apoptosis, and lessened ATP production. Moreover, mRNA vaccine significantly increases de novo lipids synthesis in lipid droplets up to 96 h and alterations in biochemical composition. It seems that the lipid composition of cells returns to the normal level for a longer incubation time (14 days). In the cell nucleus, the mRNA vaccine does not produce statistically significant changes.

## 1 Introduction

The pandemic outbreak in 2019 generating acute respiratory syndrome caused 494,587,638 confirmed cases of COVID-19, including 6,170,283 deaths, reported by WHO.^1^ Pharmaceutical companies including Pfizer/BioNTech in 2020 prepared vaccines based on mRNA technology. The Pfizer/BioNTech vaccine (BNT162b2) was reported to be more than 90% effective against COVID-19.^2^ As of 5 April 2022, a total of 11,250,782,214 doses have been administered.^3^

The pandemic has witnessed an explosion in research examining the interplay between the immune response and the intracellular metabolic pathways that mediate it. Research in the field of immunometabolism has revealed that similar mechanisms regulate the host response to infection, autoimmunity, and cancer. The new method of Raman imaging presented in this paper contributes to a better understanding of pathways of our immune responses, recognizes metabolites that regulate these pathways, and suggests how to optimize mRNA technology to stimulate the adaptive immune system.

We show that the key molecule in immunometabolism is cytochrome *c*. Cytochrome *c* plays a role as a key protein that is required for maintaining life (respiration) and cell death (apoptosis). Until now, we do not know exactly if cytochrome *c* itself controls life (respiration) and death (apoptosis) processes or whether there are other mechanisms caused by a release of cytochrome *c* from the mitochondria to the cytoplasm.

Dysregulation of oxidative phosphorylation and apoptosis in cells of the immune system can have essential consequences, which may result in diseases including cancer and autoimmunity. This paper summarizes our current understanding of the role of cytochrome *c* in cancer and its influence on immune responses to point out future directions of research.

In this paper, we will study the effect of the COVID-19 mRNA vaccine on the human lung epithelial cancer cells in the respiratory system by using a novel noninvasive tool of Raman imaging. Here, we demonstrate that Raman imaging gives new insight into the basic mechanisms of cancer pathology and the effect of mRNA vaccines on specific organelles in the *in vitro* cells.

Raman spectroscopy and imaging provide a quantitative and noninvasive method to probe intracellular changes without the need for exogenous labeling. Conventional methods of molecular biology require the destruction of cell membranes to extract intracellular components for studying the biochemical changes inside specific organelles. In Raman imaging, we do not need to break cells to learn about the biochemical composition of intracellular organelles. Visualization of alterations in biochemical composition in separate organelles is extremely valuable to monitor molecular mechanisms in cancer development and the mechanisms of infections. Until now, no technology has proven to be effective for detecting a concentration of specific chemical compounds in separate living cell organelles. Therefore, existing analytical technologies cannot detect the full extent of biolocalization of different chemical compounds inside and outside specific organelles.

Cancer diseases are the most serious cause of death, exceeding heart disease, strokes, pneumonia, and COVID-19. There is no vaccine against most cancers; the rapid development of mRNA vaccines may help in the development of anticancer vaccines shortly.

Here, we will show for the first time that Raman imaging allows for quantitative and noninvasive monitoring of biochemical compositions in specific organelles of the human lung epithelial cancer cell in response to mRNA vaccine. We will compare the effect of mRNA on cancer lung cells with that of cancer itself (control). According to our best knowledge, the mRNA vaccine has not been clinically tested for cancer patients. Therefore, this contribution will help monitor responses in host lung cells similar to a viral infection because the incubation with the COVID-19 mRNA vaccine mimics some mechanisms of COVID-19 infection. Of course, it has to be remembered that instead of the whole virus, only one protein S, essential for the immune response, is injected without COVID-19 virus replications.

It is important to monitor the biodistribution and location of metabolites upon mRNA vaccines in human host cells *in vitro* and in appropriate experimental animal models. Visualization of chemical alterations in single cells upon delivery of mRNA vaccines would help evaluate the efficacy of candidate formulations and aid their rational design for preclinical and translational studies.

We will monitor the effect of the mRNA vaccine on the biodistribution of different chemical components, particularly cytochrome *c*, in the specific organelles of a cell: nucleus, mitochondria, lipid droplets, cytoplasm, and membrane. In this article, we will explore alterations in reduction–oxidation pathways related to cytochrome *c* in human lung cancer cells upon incubation *in vitro* with the COVID-19 mRNA vaccine.

We will demonstrate that Raman spectroscopy and Raman imaging are competitive clinical diagnostic tools for cancer diseases linked to mitochondrial dysfunction and are a prerequisite for successful pharmacotherapy of cancer. The strength of our approach is that results on the biology of pulmonary cells are of great interest for patients recovering from COVID-19 because they sometimes suffer from postinfection effects of the respiratory system.

## 2 Materials and Methods

### 2.1 Reagents

Cytochrome *c* (C 2506) was purchased from Sigma-Aldrich (Poland).

### 2.2 Cell Culture, Incubation with Vaccine, and Preparation for Raman Imaging

The human lung carcinoma cell line A549 (ATCC CCL-185) was purchased from the American Type Culture Collection (ATCC). The A549 cells were maintained in F-12K medium (ATCC 30-2004) supplemented with 10% fetal bovine serum (ATCC 30-2020) without antibiotics in a humidified incubator at 37 °C under 5% CO_2_ atmosphere. For Raman imaging, cells were seeded on a CaF_2_ window (Crystran Ltd., CaF_2_ Raman-grade optically polished window 25 mm dia × 1 mm thick, no. CAFP25-1R) in a 35 mm Petri dish at a density of 5 × 10^4^ cells per Petri dish. On the following day, the culture medium was replaced with the culture medium (F-12K medium (ATCC 30-2004) supplemented with 10% fetal bovine serum) supplemented with 1 or 60 μL of diluted COVID-19 mRNA vaccine per 1 mL of medium. We diluted the COVID-19 mRNA vaccine (Pfizer/BioNTech (BNT162b2)) in 1.8 mL of 0.9% sodium chloride.^4^ The real dose of the diluted vaccine that is administered to patients is equal to 0.3 mL, corresponding to 30 μg of mRNA per dose. In our experiments, the total volume of used Petri dishes was 3 mL; so that gives us doses of 0.3 and 18 μg of mRNA per Petri dish (1 or 60 μL of diluted vaccine per 1 mL of medium). The incubation time with the vaccine was 1, 24, 72, 96 h, and 14 days, respectively.

Before Raman examination, the growing medium was removed, the cells were fixed with 10% formalin for 10 min, and kept in phosphate-buffered saline (PBS, Gibco no. 10010023) during the experiment.

### 2.3 Raman Imaging and Spectroscopy

**Scheme 1:**
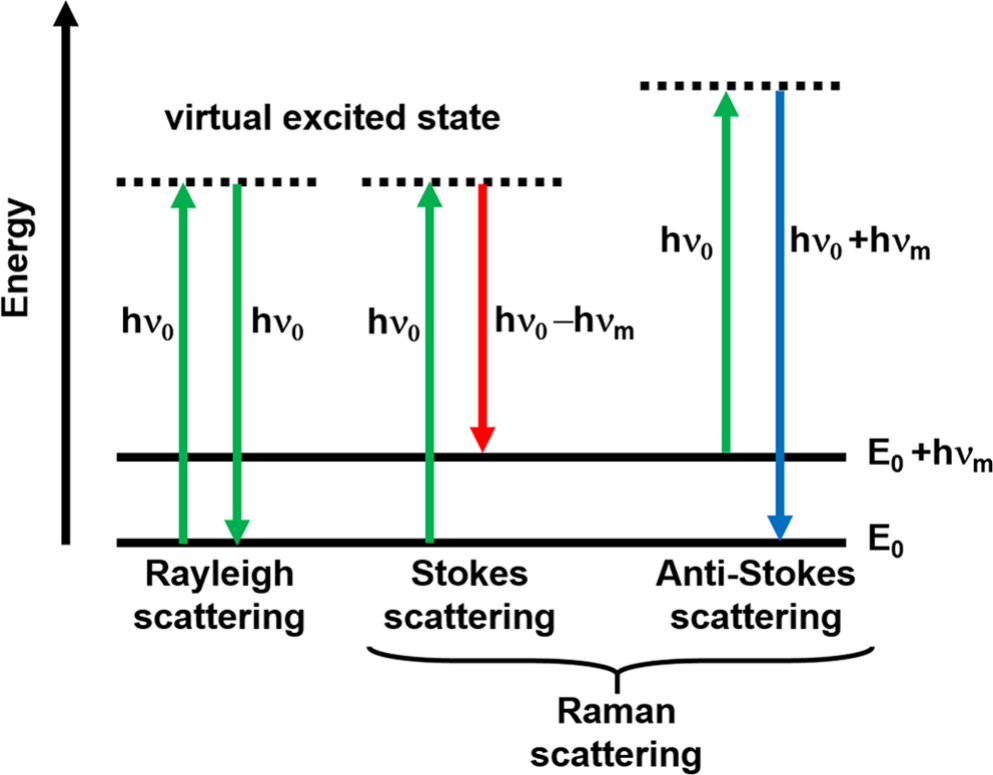
Schematic Presentation of Scattering Phenomena

Raman spectroscopy is an analytical method in which inelastic scattered light is used to obtain information about molecular vibrations. Raman scattering is an inelastic scattering process with a transfer of energy between the molecular vibrations (and rotations) and the scattered photons. If the molecule receives energy from the photon energy being excited to a higher vibrational, the scattered photon loses energy, and the Stokes Raman scattering occurs. Inversely, if the molecule occupying a higher vibrational state is excited and then loses energy by returning to a lower vibrational level, the scattered photon gains the corresponding energy, and Anti-Stokes Raman scattering occurs. The Stokes Raman scattering is always more intense than the Anti-Stokes component, and for this reason, we measured the Stokes signal by Raman spectroscopy. The Rayleigh, Stokes, and Anti-Stokes Raman Scattering processes are presented in Scheme 1.

**Scheme 2:**
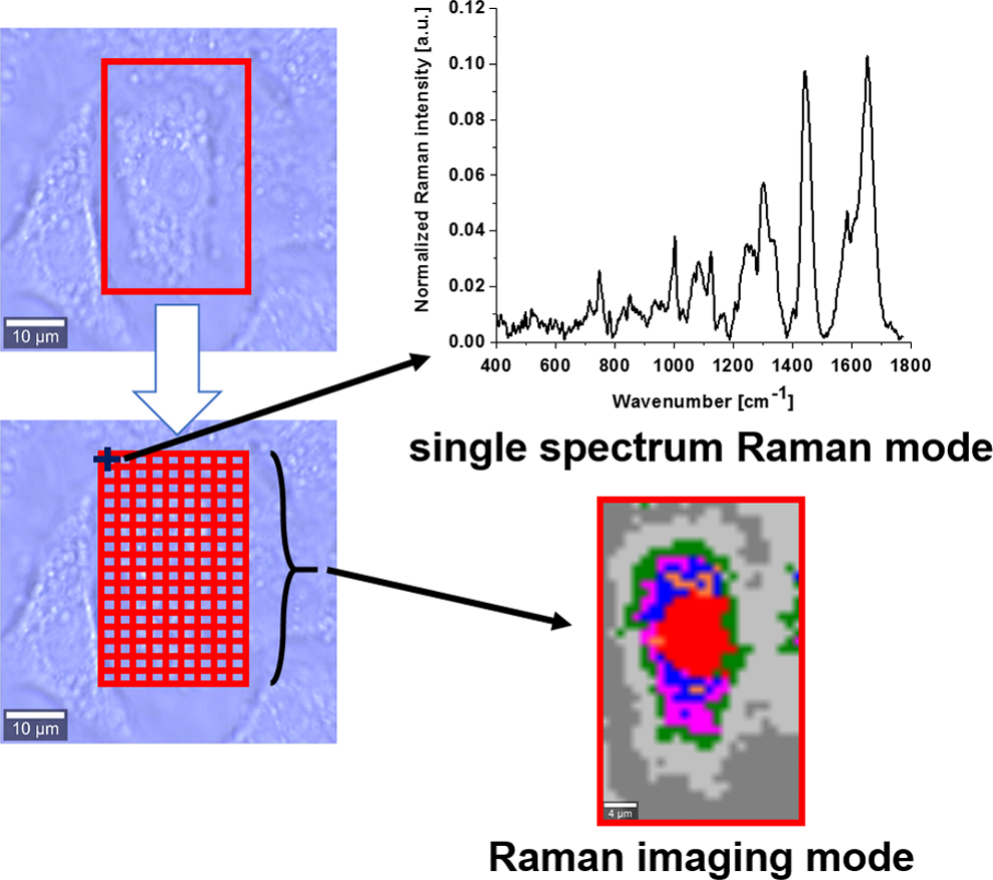
Data Acquisition for a Single Point in a Cell Spectrum and Raman Imaging of a Whole Cell (A549)

Raman imaging is a technique based on Raman scattering, allowing us to measure vibrational spectra of any area. The imaging mode allows analysis of the distribution of different chemical molecules inside the sample area. Using algorithms of artificial intelligence such as Cluster Analysis (see Section 2.4) based on two-dimensional (2D) data makes it possible to create Raman maps to visualize a cell’s organelles: nucleus, mitochondria, lipid structures, cytoplasm, and cell membrane and learn about their biocomposition. Scheme 2 illustrates the idea of data acquisition for a single cell spectrum and Raman imaging.

Raman spectra and images were recorded using a confocal Raman microscope (WITec (alpha 300 RSA+), Ulm, Germany) in the Laboratory of Laser Molecular Spectroscopy, Lodz University of Technology, Poland. The excitation laser at 532 nm was focused on the sample to the laser spot of 1 μm with the microscope (Olympus Düsseldorf, Germany) with A 40× water immersion objective (Zeiss, W Plan-Apochromat 40×/1.0 DIC M27 (FWD = 2.5 mm), vis–IR) via an optical fiber with a diameter of 50 μm. The laser excitation power was 10 mW. Raman images were recorded with a spatial resolution of 1 μm × 1 μm and a collection time of 0.5 and 1 s for Raman images with a UHTS (ultrahigh-throughput screening) monochromator (WITec, Ulm, Germany) and a thermoelectrically cooled CCD camera ANDOR Newton DU970N-UVB-353 (EMCCD (Electron Multiplying Charge Coupled Device, Andor Technology, Belfast, Northern Ireland) chip with 1600 × 200 pixel format, 16 μm dimension each) at −60 °C with full vertical binning. The Raman spectrometer was calibrated before the measurements using a silica plate with a maximum peak at 520.7 cm^–1^.

### 2.4 Data Processing and Cluster Analysis

WITec Project Plus software was used to collect and process the Raman data. To refine background subtraction and normalization (model: divided by vector norm), we used Origin software. Normalization divided by vector norm means to divide the curve (Raman spectrum) by the norm of the *Y* values (Raman intensities for each wavenumber). Spectroscopic data were analyzed by using the Cluster Analysis method. Details are given in our previous paper.^5^ The normalization was performed for two regions: fingerprint region (370–1770 cm^–1^) and high-frequency region (2700–3100 cm^–1^) separately. The Raman maps presented in the manuscript were constructed based on the number of clusters of 7. Each cluster is characterized by a different average Raman spectrum and describes the inhomogeneous distribution of chemical components of different organelles within the cell.

## 3 Results

To properly address alterations in single lung cells upon incubation with the COVID-19 mRNA vaccine, we systematically investigated how Raman spectroscopy and Raman imaging monitor responses to the vaccine in specific organelles.

**Figure 1:**
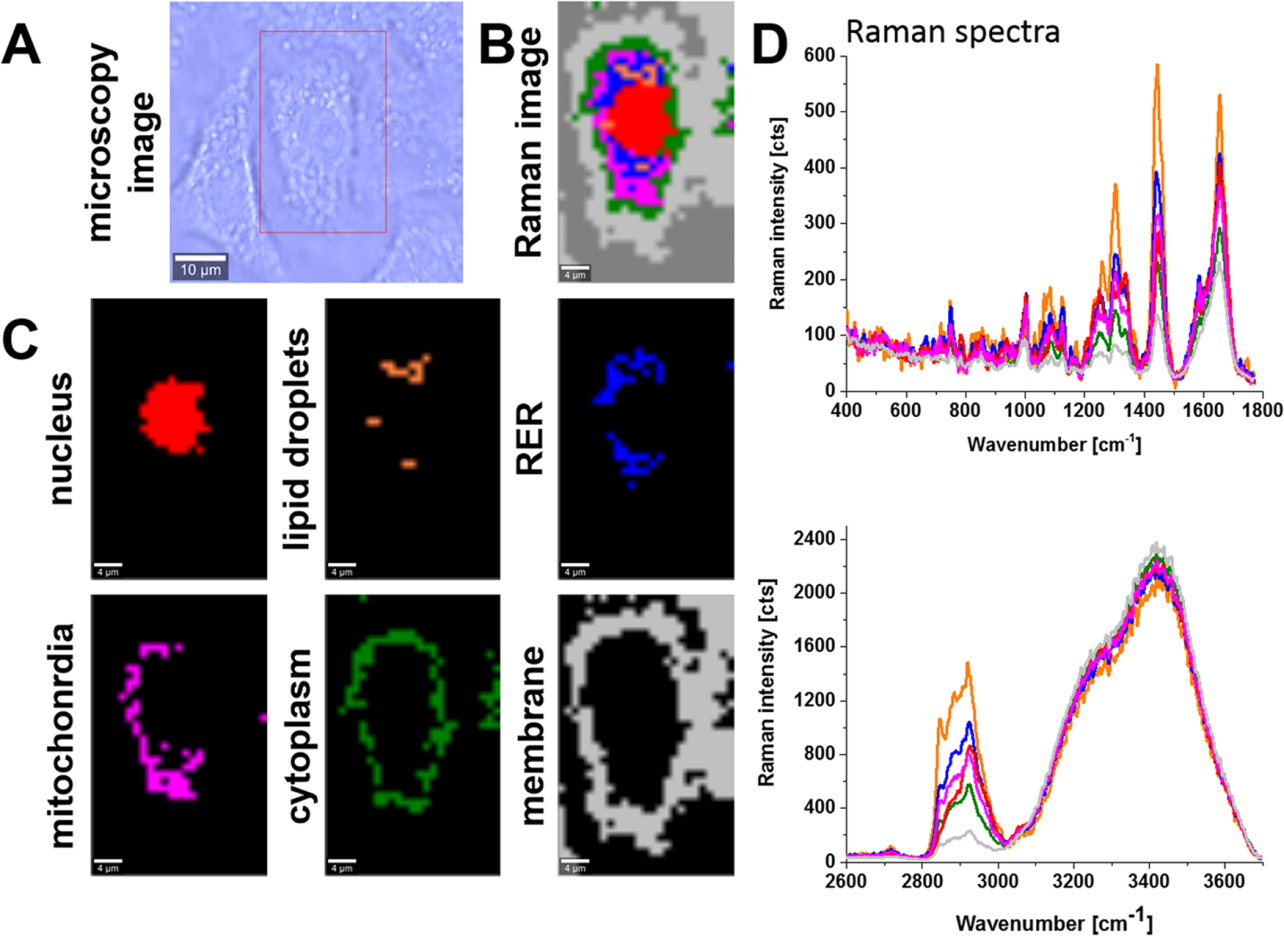
Microscopy image (A), Raman image of human lung carcinoma (A549) cell (25 μm × 40 μm, resolution 1.0 μm) incubated with COVID-19 mRNA vaccine (dose 60 μL) for 96 h (B) and Raman images of specific organelles: nucleus (red), lipid droplets (orange), rough endoplasmic reticulum (RER, blue), mitochondria (magenta), cytoplasm (green), and membrane (light gray) (C) with corresponding Raman spectra (D) at 532 nm.

Figure 1 shows the Raman image of a typical cell of highly aggressive lung cancer incubated with mRNA vaccine for a dose of 60 μL for the period of incubation of 96 h and Raman images of specific organelles. The Raman images were created by K-means cluster analysis (seven clusters). The blue, orange, magenta, red, green, and light gray color represent lipids in lipid droplets and rough endoplasmic reticulum, lipid droplets filled with triacylglycerols of monounsaturated type (TAG), mitochondria, nucleus, cytoplasm, and membrane, respectively. The dark gray color corresponds to the area out of the cell.

To track alterations in human lung cancer cells upon incubation with the COVID-19 vaccine, we systematically investigated how the average Raman spectra in each organelle of cells incubated with mRNA and control cells without mRNA incubation.

### 3.1 Mitochondria-mRNA

**Figure 2:**
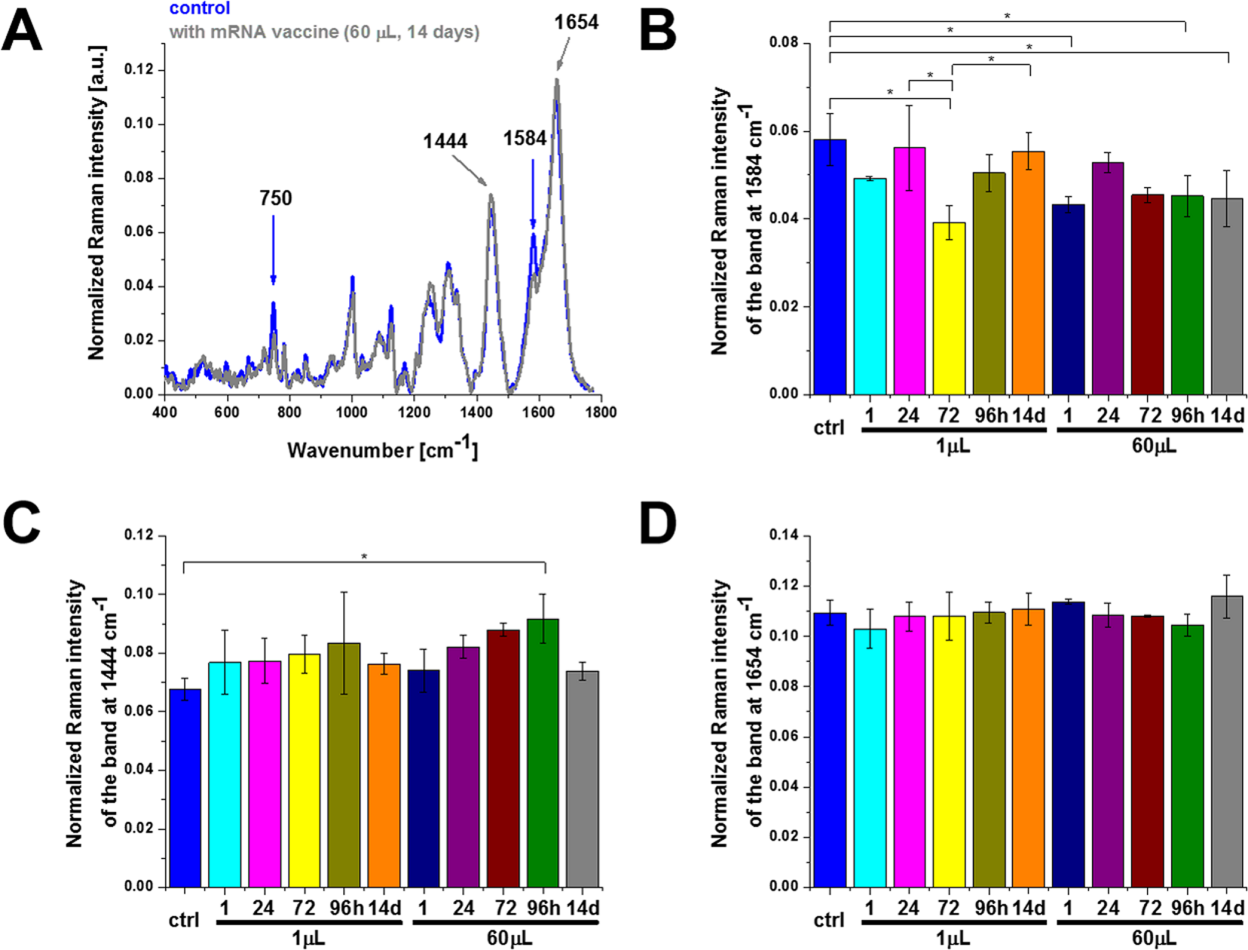
Effect of the COVID-19 mRNA vaccine on mitochondria in human lung carcinoma (A549) cells (A) upon incubation for 1, 24, 72, 96 h, and 14 days for 1 and 60 μL doses at 1584 cm^–1^ (B), 1444 cm^–1^ (C), and 1654 cm^–1^ (D) (number of cells: 3, number of Raman spectra for each single cell: minimum 1600, excitation wavelength: 532 nm, laser power: 10 mW, integration time: 1.0 s). The statistical significance was calculated with the one-way ANOVA using the Tukey test; asterisk * denotes that the differences are statistically significant; *p*-value ≤ 0.05.

Figure 2 shows the effect of the COVID-19 mRNA vaccine on mitochondria in human lung carcinoma (A549) cells upon incubation for 1, 24, 72, 96 h, and 14 days for 1 and 60 μL doses at 1584, 1444, and 1654 cm^–1^.

Detailed inspection of Figure 2 demonstrates that the most significant changes occur at 750 and 1584 cm^–1^ corresponding to cytochrome *c*(6) and at 1444 cm^–1^ corresponding to saturated C–H bending vibrations of cholesterol and other lipids.^7^ Let us first focus on cytochrome *c*. Briefly, there is a broad family of cytochromes that are classified based on the lowest electronic energy absorption band in their reduced state, such as cytochrome P450 (450 nm), cytochrome *c* (Q-band 550 nm), cytochromes *b* (≈565 nm), and cytochromes a (605 nm). We used laser excitation with a wavelength at 532 nm, whose energy coincides with the electron absorption of the Q-band. This resonance leads to greatly enhanced intensity of the Raman scattering, which is called the Resonance Raman Effect (RRE). RRE can be obtained when the incident laser radiation is at a frequency near the frequency of an electronic transition of the molecule of interest. RRE amplification facilitates the study of cytochrome *c*, which is present at low concentrations under physiological conditions of humans. The cytochrome *c* is localized at the internal mitochondrial membranes between the complex known as complex III (sometimes also called Coenzyme Q–Cyt *c* reductase or cytochrome *bc1* complex) and complex IV (also called cytochrome *c* oxidase). Cytochrome *c* plays a key role in the electron transport chain of the oxidative phosphorylation (respiration) process. The oxidized cytochrome *c* (cyt *c* Fe^3+^) is reduced to cytochrome *c* (cyt *c* Fe^2+^) by the electron obtained from complex III. The reduced cytochrome *c* passes an electron to the copper binuclear center of complex IV, being oxidized back to cytochrome *c* (cyt *c* Fe^3+^). Complex IV, which is the final complex in the electron transport chain, contains two cytochromes *a* and *a3* and two copper centers. The electron transport chain is followed by an ATP synthase, which is often called complex V in the electron transport chain. Therefore, under normal conditions, cytochrome *c* in the mitochondrion exists in the oxidized form. Under pathological conditions, metabolic processes in mitochondrion result in downregulation of the transfer between cytochrome *c* and complex IV leading to an increase of the reduced form of cytochrome in tissue and blood. Raman signals of the oxidized form are significantly lower than those of the reduced form of cytochrome *c*.^5^ Therefore, Raman spectra can be used as markers of the redox status of cytochrome *c*.

The peak at 1584 cm^–1^ in Figure 2 represents the “redox state Raman marker” of cytochrome *c*. Recently, we demonstrated that this Raman vibration can serve as a sensitive indicator of oxidized and reduced forms of cytochrome *c*.^5,6,8^ It indicates that the Raman peak at 1584 cm^–1^ can be used as a marker to explore apoptosis and oxidative phosphorylation in mitochondria.^6,9^ This band reflects the dual activity of cytochrome *c* in life and death processes: apoptosis and oxidative phosphorylation. The balance between the proliferation of cancer cells (oxidative phosphorylation) and cell death (apoptosis) determines the rate of cancer development and cancer aggressiveness.^6,10^ The Raman signal of a single cell at 1584 cm^–1^ depends on cytochrome *c* concentration (which also depends on the number of mitochondria in a cell) and the redox state (oxidized or reduced forms). The Raman intensity of the oxidized form is much weaker than that of the reduced form.^5,6,8,9^

Inside a normal mitochondrion, cytochrome *c* exists in the oxidized form. Dysfunction of mitochondrion associated with several malignancies, including cancer or virus infections blocks the transfer of electrons between complexes III and IV of the electron transport chain, resulting in lower efficiency of the oxidative phosphorylation (respiration) process and lower ATP synthesis. This results in a change of the redox status of cytochrome *c* from the oxidized (cyt *c* Fe^3+^) to the reduced form (cyt *c* Fe^2+^).^5^ Cytochrome *c* not only acts as a mitochondrial charge carrier that transfers electrons between complexes III and IV but also activates the caspase cascade when cytochrome *c* is released from the intermembrane space of the mitochondrion to the cytoplasm. The released cytochrome *c* interacts with apoptosis-protease activating factor 1 (Apaf-1), triggering the caspase cascade in the cell and induces an inflammatory response in the immune system. The activated caspases ultimately lead to cell apoptosis. Therefore, it was suggested that cytochrome *c* may play the role of a universal DAMP molecule (damage-associated molecular patterns) that informs the immune system of infection danger in cells or tissues.^11^ DAMP molecules are released from damaged or dead cells and activate the innate immune system by interacting with pattern recognition receptors (PRRs).^12^ Therefore, cytochrome *c* plays a double function: it warns the immune system and contributes to the host’s defense and also promotes pathological inflammatory responses. Controlling cell death by apoptosis is necessary for the normal functioning of the immune system^13^ as well as is very important in natural mechanisms protecting against cancer. When less cytochrome *c* is released as DAMP molecules from the damaged or dying cells, the innate immune system is not activated to the sufficient level to protect the body by interacting with PRRs.

A similar mechanism may play a key role in the adaptive immune system because it is reported that cytokines play an important role in adaptive immunity.^14,15^ CD4^+^ helper T cells (Th) can be divided into two subgroups, type I helper T lymphocytes (Thl) and type II helper T cells (Th2).^14^

We provide evidence that the balance between apoptosis and oxidative phosphorylation is regulated by interactions between cytochrome *c* and cardiolipin.^5^ Cardiolipin-bound cytochrome *c* probably does not participate in electron shuttling of the respiratory chain,^16^ leading to the production of a reduced form of cytochrome *c* resulting in lower efficiency of respiration (oxidative phosphorylation) and lessened ATP production. The reduced form of cytochrome bound to cardiolipin cannot induce the caspase and apoptosis process.^5,16^

The intensity of the Raman signal at 1584 cm^–1^ corresponding to the concentration of cytochrome *c* in mitochondria depends on four factors: (a) redox status (oxidized or reduced form of cytochrome *c*), (b) release of cytochrome *c* to the cytoplasm, (c) number of cytochrome *c* molecules in mitochondria that depends on the number of mitochondria in a single cell, (d) transformation of cytochrome *c* function from electron carrier in the electron transport chain into the peroxidase activity, i.e., it catalyzes the oxidation of organic substrates by H_2_O_2_. This peroxidase function plays a key role during apoptosis.^17^

The peroxidase activity decreases the Raman signal at 1584 cm^–1^ of cytochrome *c* because the oxidized Fe^3+^ cytochrome *c* in mitochondria upon interactions with cardiolipin monitors the transformation of Fe^3+^ cytochrome *c* into iron-oxo (ferryl) intermediates called Compound I and Compound II, which are (unprotonated) Fe^IV^=O or (protonated) Fe^IV^–OH.^17−21^

As the Raman signals of the reduced form are significantly higher than those of the oxidized form of cytochrome *c*,^5^ one can state that the Raman intensity of the band at 1584 cm^–1^ in Figure 2A,B represents an oxidized form of cytochrome (cyt *c* Fe^3+^) both for the control and the cells upon incubation with the COVID-19 mRNA vaccine. In the following paragraph, we demonstrate that there is no release of cytochrome *c* to the cytoplasm. It indicates that the concentration of cytochrome *c* (cyt *c* Fe^3+^) must be determined by the factor (c) the number of mitochondria in a single cell or (d) peroxidase activity. Both factors (c) and (d) result in reduced activities of oxidative phosphorylation and apoptosis. The results in Figure 2A,B show the Raman signal at 1584 cm^–1^ upon incubation with the mRNA vaccine decreases when compared with the control (without the mRNA vaccine, blue color), indicating reduced metabolic activities.

In view of the results presented in Figure 2, one can state that the mRNA vaccine does not block the transfer of electrons between complexes III and IV of the respiratory chain but results in lower efficiency of oxidative phosphorylation and ATP synthesis and reduced apoptosis.

It is worth noticing that a similar effect of downregulation of cytochrome *c* concentration was reported for brain cancer cells vs cancer aggressiveness.^6^ We showed that the Raman signal of the band at 1584 cm^–1^ related to the concentration of cytochrome *c* in mitochondria of a single cell *in vitro* decreases with brain tumor aggressiveness.^6,9^

### 3.2 Cytoplasm-mRNA

**Figure 3:**
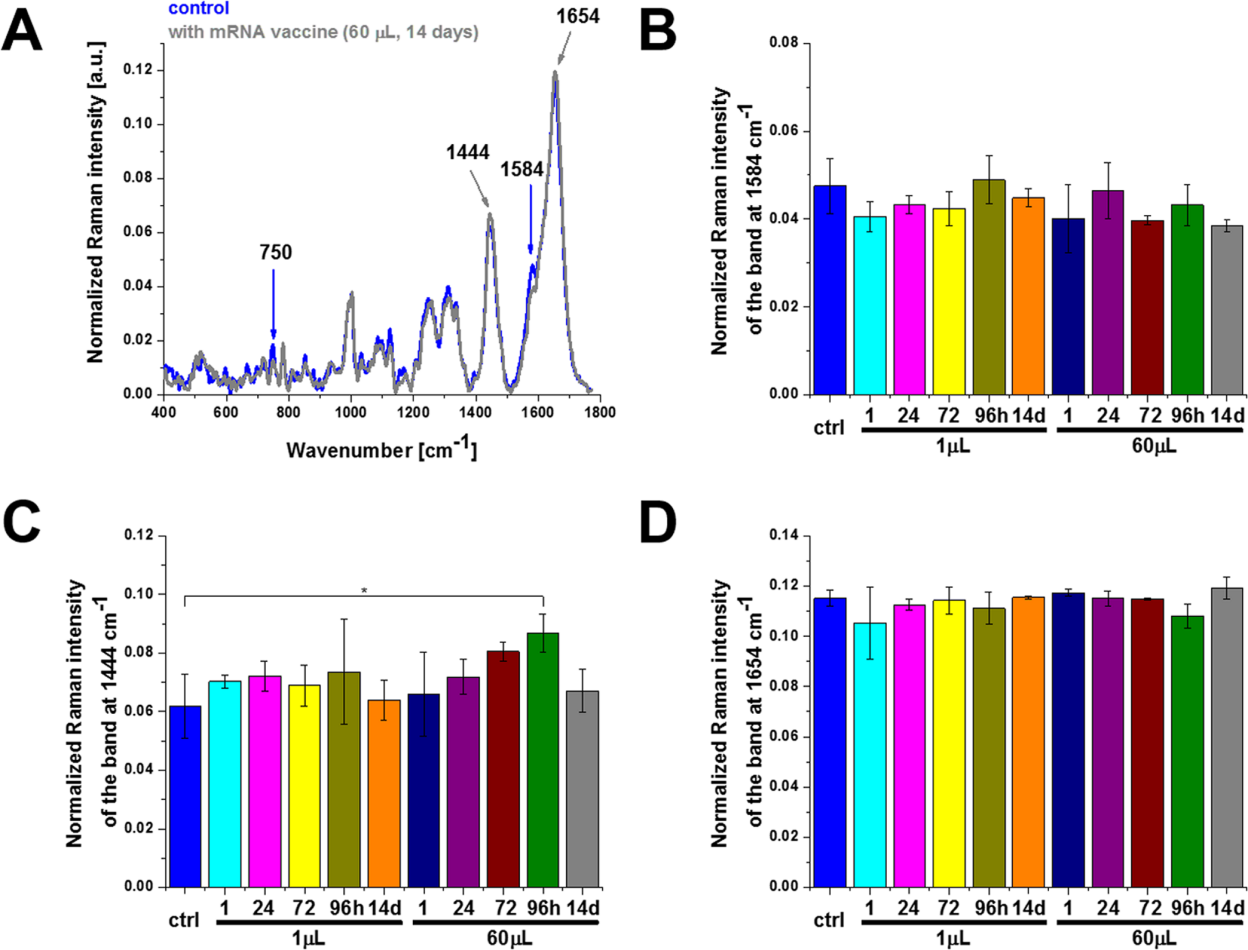
Effect of the COVID-19 mRNA vaccine on the cytoplasm in human lung carcinoma (A549) cells (A) upon incubation for 1, 24, 72, 96 h, and 14 days for 1 and 60 μL doses at 1584 (B), 1444 (C), and 1654 cm^–1^ (D) (number of cells: 3, number of Raman spectra for each single cell: minimum 1600, excitation wavelength: 532 nm, laser power: 10 mW, integration time: 1.0 s). The one-way ANOVA using the Tukey test was used to calculate the significance value; asterisk * denotes that the differences are statistically significant; *p*-value ≤ 0.05.

To check if the observed lower concentration of cytochrome *c* in mitochondria is related to the release of cytochrome *c* (apoptosis) to the cytoplasm, we studied the localization of cytochrome *c* in the cytoplasm. Figure 3 shows the effect of the COVID-19 mRNA vaccine on the Raman spectra in the cytoplasm of human lung carcinoma (A549) by Raman imaging compared with those of the control cells. Figure 3 shows a comparison of the average Raman for cytoplasm at 532 nm excitation with and without the COVID-19 mRNA vaccine. One can see that the Raman signal at 1584 cm^–1^ in the cytoplasm (Figure 3B) is the same for control cells and those incubated with mRNA within statistical significance at *p*-value ≤ 0.05. It means that the lower concentration of cytochrome *c* in mitochondria is not related to the release to the cytoplasm. In view of this result from Figure 3B, one can state that the mRNA does not activate additional apoptosis via cytochrome *c* release and does not act as a cytoplasmic apoptosis-triggering agent.

### 3.3 Lipid Droples-mRNA and Rough Endoplasmic Reticulum-mRNA

**Figure 4:**
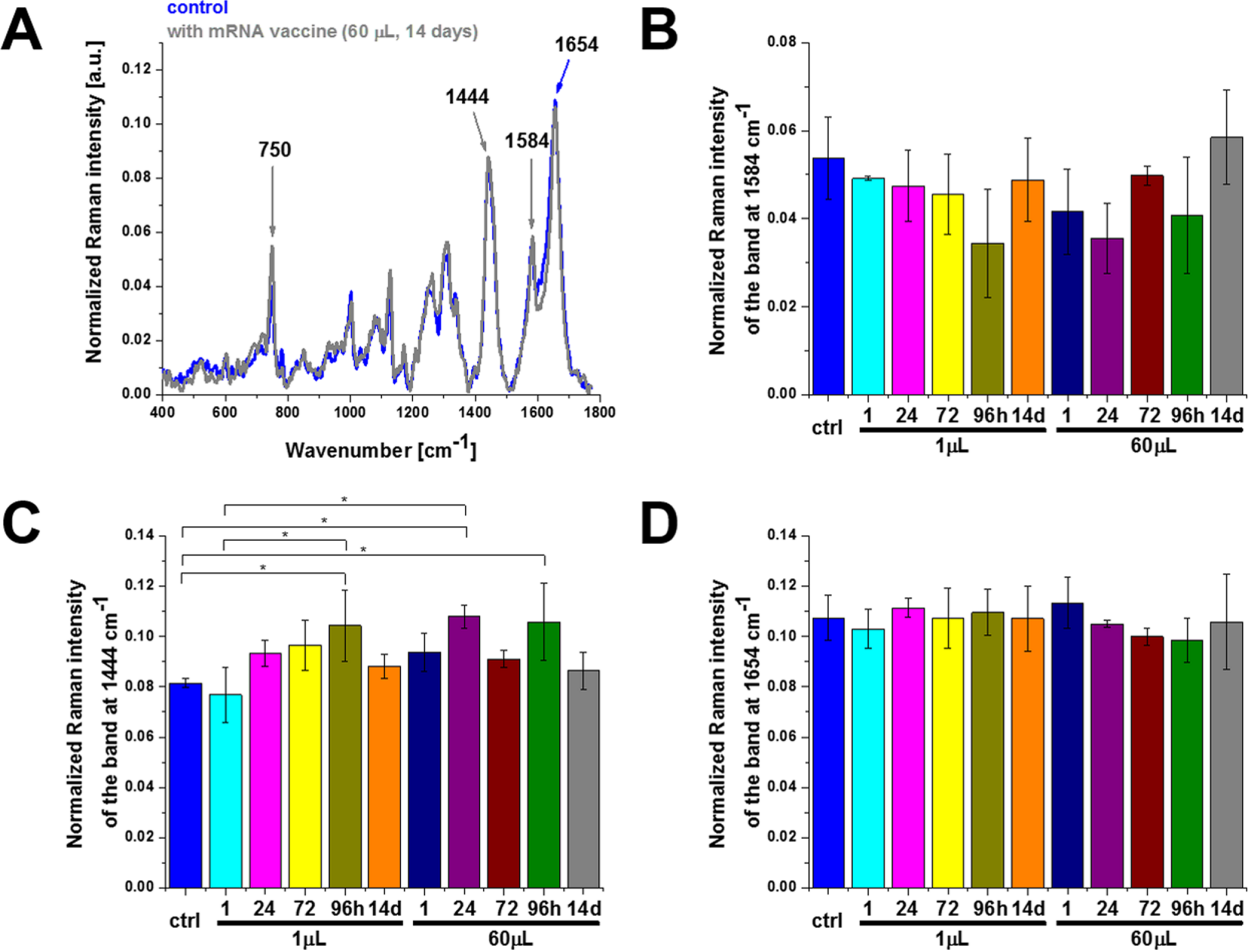
Effect of the COVID-19 mRNA vaccine on lipid droplets in human lung carcinoma (A549) cells (A) upon incubation for 1, 24, 72, 96 h, and 14 days for 1 and 60 μL doses at 1584 cm^–1^ (B), 1444 cm^–1^ (C), and 1654 cm^–1^ (D) (number of cells: 3, number of Raman spectra for each single cell: minimum 1600, excitation wavelength: 532 nm, laser power: 10 mW, integration time: 1.0 s). The one-way ANOVA using the Tukey test was used to calculate the significance value; asterisk * denotes that the differences are statistically significant; *p*-value ≤ 0.05.

**Figure 5:**
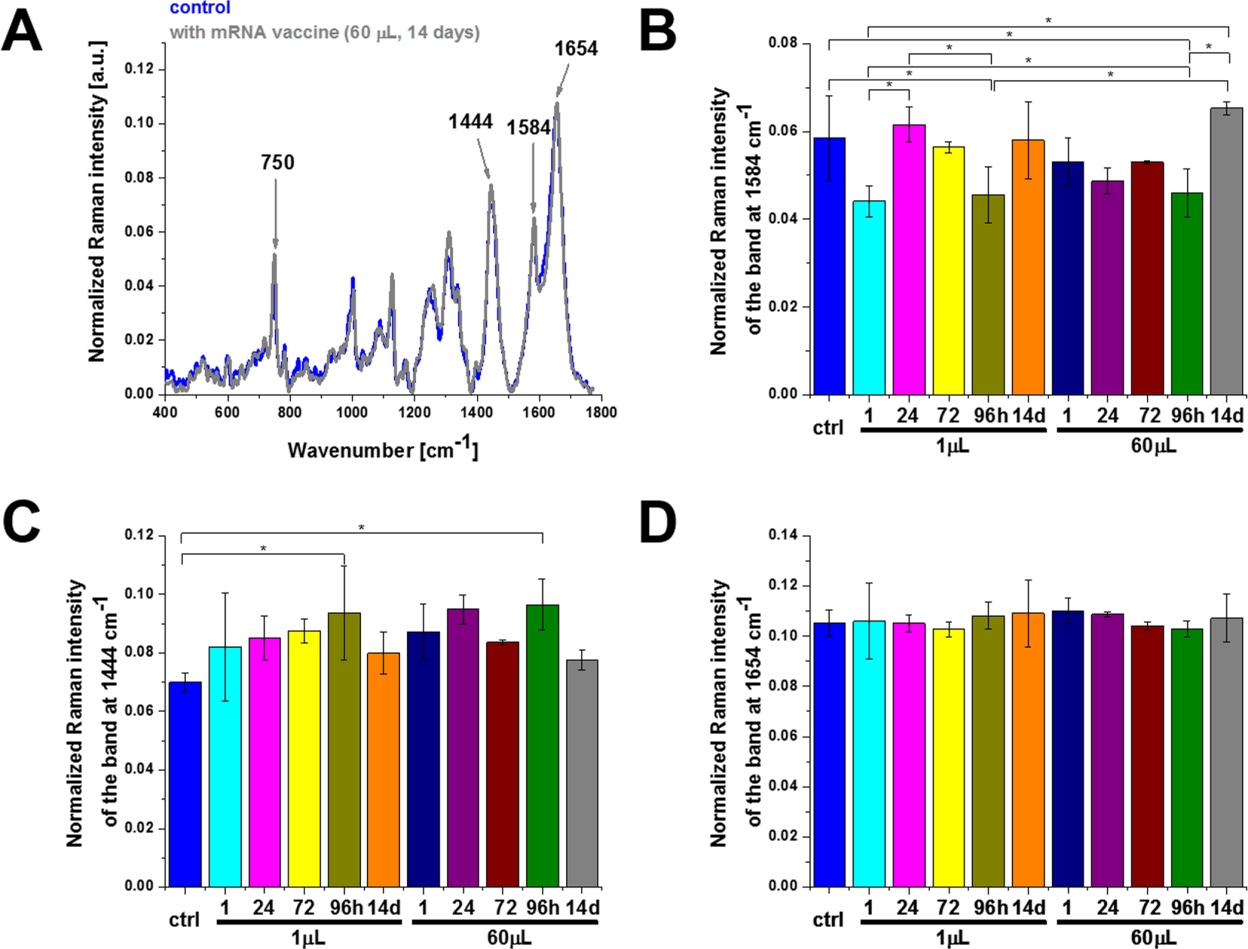
Effect of the COVID-19 mRNA vaccine on rough endoplasmic reticulum in human lung carcinoma (A549) cells (A) upon incubation for 1, 24, 72, 96 h, and 14 days for 1 and 60 μL doses at 1584 cm^–1^ (B), 1444 cm^–1^ (C), and 1654 cm^–1^ (D) (number of cells: 3, number of Raman spectra for each single cell: minimum 1600, excitation wavelength: 532 nm, laser power: 10 mW, integration time: 1.0 s). The one-way ANOVA using the Tukey test was used to calculate the significance value; asterisk * denotes that the differences are statistically significant; *p*-value ≤ 0.05.

Significant changes in the cytochrome *c* concentration are also observed in other organelles. Similar to mitochondria, alterations in the biochemical composition of cytochrome *c* reflected by the band at 1584 cm^–1^ are also observed in lipid droplets and lipid structures of the rough endoplasmic reticulum (ER) presented in Figures 4 and 5. Rough ER contains ribosomes that are responsible for protein synthesis (mRNA translation). Therefore, ribosomes are the sites where the protein synthesis for mRNA vaccines occurs. Ribosomes are too small to be seen separately in the ER at the resolution offered by Raman imaging.

Now, we focus on lipid synthesis upon incubation with the mRNA vaccine. Figures 2–5 show that the Raman bands at 1444 cm^–1^ are significantly modified upon incubation with mRNA. Indeed, the Raman signal at 1444 cm^–1^ corresponding to C–H bending vibrations of lipids in lipid droplets (orange color in Figure 1) and in the rough endoplasmic reticulum (blue color in Figure 1) clearly increases upon incubation with the COVID-19 mRNA vaccine. The results indicate that the mRNA vaccine upregulates de novo lipid synthesis up to 96 h. It seems that the lipid composition of cells returns to the normal level for a longer incubation time (14 days).

### 3.4 Nucleus-mRNA

**Figure 6:**
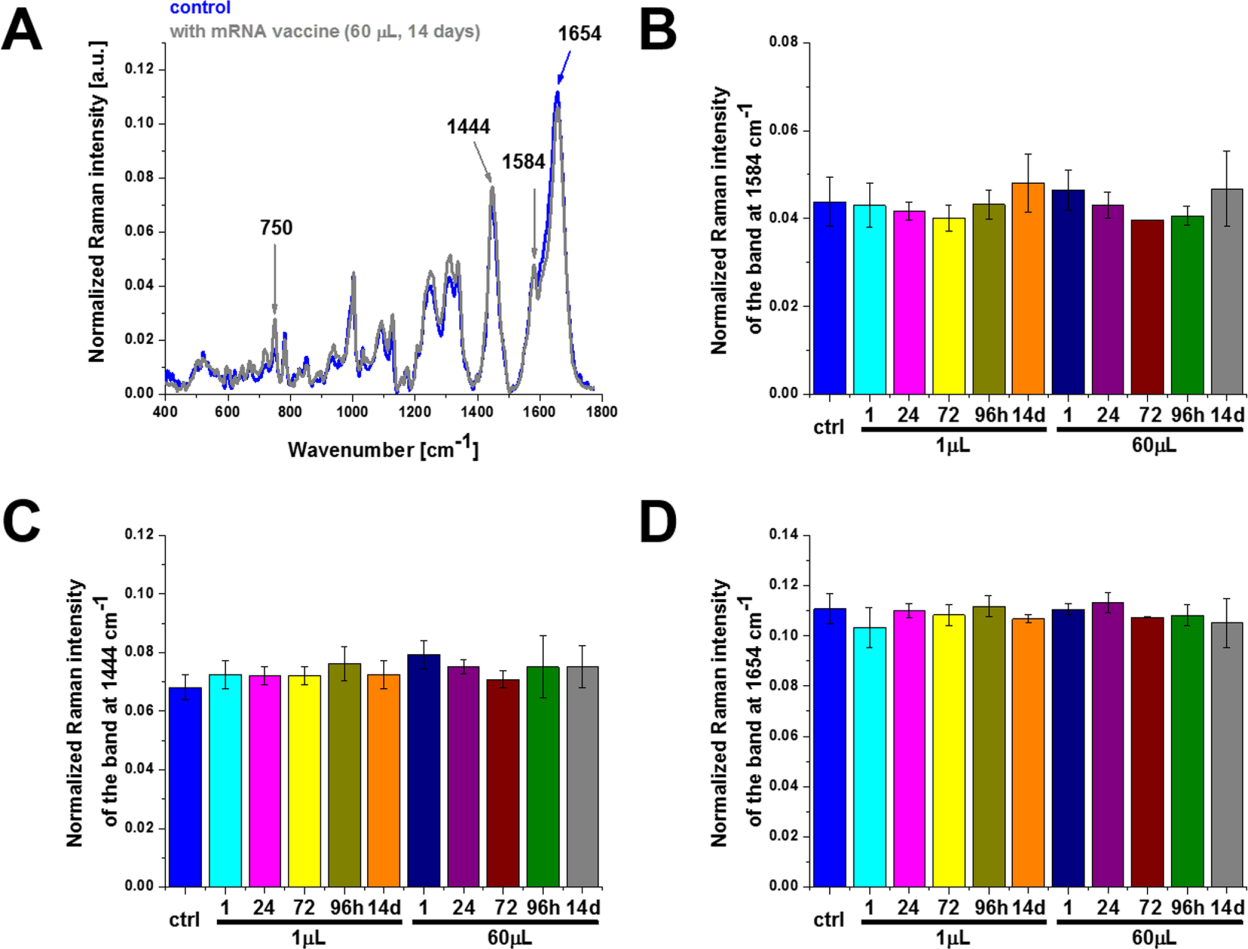
Effect of the COVID-19 mRNA vaccine on the nucleus in human lung carcinoma (A549) cells (A) upon incubation for 1, 24, 72, 96 h, and 14 days for 1 and 60 μL doses at 1584 cm^–1^ (B), 1444 cm^–1^ (C), and 1654 cm^–1^ (D) (number of cells: 3, number of Raman spectra for each single cell: minimum 1600, excitation wavelength: 532 nm, laser power: 10 mW, integration time: 1.0 s). The one-way ANOVA using the Tukey test was used to calculate the significance value; asterisk * denotes that the differences are statistically significant; *p*-value ≤ 0.05.

It is claimed that mRNA vaccine does not enter the nucleus of the cell where our DNA (genetic material) is kept.^22^ The explanation is the following: (1) The cell breaks down and gets rid of the mRNA soon after it finishes the translation procedure, (2) neither the coronavirus nor the RNA vaccines have a reverse transcriptase. Therefore, RNA vaccines cannot produce DNA molecules, (3) the cells keep their compartments well-separated, and mRNAs cannot travel from the cytoplasm to the nucleus because the lifetime of mRNA molecules is short. The mRNA is a short-lived molecule and is degraded after a few hours; (4) reported clinical studies have shown no sign of DNA modification so far. These facts suggest that vaccines do not alter our genome. Figure 6 shows the results obtained for the nucleus without and upon incubation with the vaccine by Raman imaging. Our results support the conclusion that the mRNA vaccine does not enter the DNA of the cell, as we observe no statistically significant changes in the Raman signals.

### 3.5 Membrane-mRNA

As is well-known, professional antigen-presenting cells (APCs) play a crucial role in initiating immune responses and are mainly represented by professional APC dendritic cells. However, under pathological conditions, epithelial cells also act as nonprofessional APCs, thereby regulating immune responses at the site of exposure. Therefore, it is interesting to monitor alterations at the surface of the cell membranes of lung epithelial cells upon incubation with mRNA.

**Figure 7:**
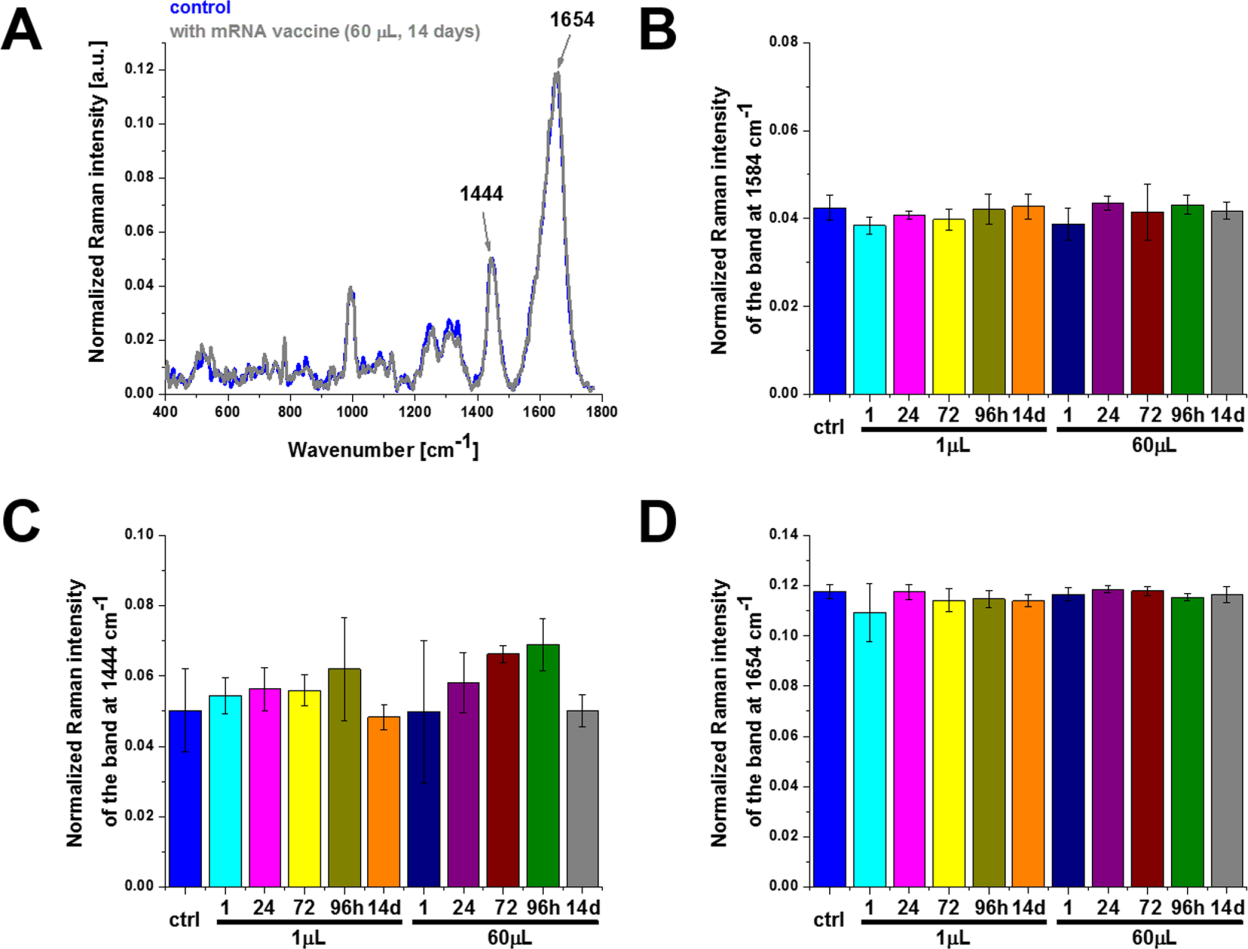
Effect of the COVID-19 mRNA vaccine on a membrane in human lung carcinoma (A549) cells (A) upon incubation for 1, 24, 72, 96 h, and 14 days for 1 and 60 μL doses at 1584 (B), 1444 (C), and 1654 cm^–1^ (D) (number of cells: 3, number of Raman spectra for each single cell: minimum 1600, excitation wavelength: 532 nm, laser power: 10 mW, integration time: 1.0 s). The one-way ANOVA using the Tukey test was used to calculate the significance value; asterisk * denotes that the differences are statistically significant; *p*-value ≤ 0.05.

Figure 7 shows the effect of incubation with the COVID-19 mRNA vaccine compared with the control cells without mRNA at the surface of the membrane. One can see that the cytochrome *c* activity at 1584 cm^–1^ does not change with statistical significance at *p*-value ≤ 0.05, and also the Raman signals at 1444 and 1654 cm^–1^ do not change within statistical significance.

## 4 Conclusions

The effect of the COVID-19 mRNA vaccine on human lung carcinoma epithelial cells (A549) *in vitro* was studied by means of Raman spectroscopy and imaging. We studied biodistribution of different chemical components, particularly cytochrome *c* and lipids in the specific organelles of the cells: mitochondria, nucleus, lipid droplets, rough endoplasmic reticulum, cytoplasm, and membrane, and we observed biochemical alterations upon incubation with the COVID-19 mRNA vaccine.

Cytochrome *c* activity monitored by the Raman intensity band at 1584 cm^–1^ as a proposed redox-state Raman biomarker shows downregulation in the efficiency of oxidative phosphorylation and apoptosis after the COVID-19 mRNA vaccine incubation. Lower concentration of oxidized cytochrome *c* observed in mitochondria in human lung cancer cells upon incubation with the COVID-19 mRNA vaccine leads to reduced oxidative phosphorylation (respiration) and lessened ATP production. Incubation in the *in vitro* cells with mRNA vaccine significantly increases the de novo lipid synthesis in lipid droplets and rough endoplasmic reticulum monitored by the Raman intensity band at 1444 cm^–1^. For longer incubation time (14 days), it seems that the lipid composition of cells returns to the normal level. mRNA vaccine does not produce statistically significant changes in the nucleus.

## Data Availability

The raw data underlying the results presented in the study are available from the Lodz University of Technology Institutional Data Access. Request for access to those data should be addressed to the Head of the Laboratory of Laser Molecular Spectroscopy, Institute of Applied Radiation Chemistry, Lodz University of Technology. Data requests might be sent by email to the secretary of the Institute of Applied Radiation Chemistry: mitr@mitr.p.lodz.pl.

## References

[ref1] WHO Coronavirus (COVID-19) Dashboard | WHO Coronavirus (COVID-19) Dashboard With Vaccination Data.

[ref2] Doshi P. Covid-19 Vaccines: In the Rush for Regulatory Approval, Do We Need More Data?. BMJ 2021, 373, n1244 10.1136/bmj.n1244. 34006591

[ref3] More Than 9.6 Billion Shots Given: Covid-19 Tracker. Bloomberg.com. https://www.bloomberg.com/graphics/covid-vaccine-tracker-global-distribution/ (accessed Jan 13, 2022).

[ref4] COMIRNATY_PM_EN_262659_14-Apr-2022.Pdf. https://www.pfizer.ca/sites/default/files/202204/COMIRNATY_PM_EN_262659_14-Apr-2022.pdf (accessed May 10, 2022).

[ref5] Abramczyk H.; Brozek-Pluska B.; Kopeć M. Double Face of Cytochrome c in Cancers by Raman Imaging. Sci. Rep. 2022, 12 (1), 2120 10.1038/s41598-022-04803-0. 35136078 PMC8826388

[ref6] Abramczyk H.; Surmacki J. M.; Brozek-Pluska B.; Kopec M. Revision of Commonly Accepted Warburg Mechanism of Cancer Development: Redox-Sensitive Mitochondrial Cytochromes in Breast and Brain Cancers by Raman Imaging. Cancers 2021, 13 (11), 2599 10.3390/cancers13112599. 34073216 PMC8198470

[ref7] Movasaghi Z.; Rehman S.; Rehman I. U. Raman Spectroscopy of Biological Tissues. Appl. Spectrosc. Rev. 2007, 42 (5), 493–541. 10.1080/05704920701551530.

[ref8] Abramczyk H.; Surmacki J. M.; Brozek-Pluska B. Redox State Changes of Mitochondrial Cytochromes in Brain and Breast Cancers by Raman Spectroscopy and Imaging. J. Mol. Struct. 2022, 1252, 132134 10.1016/j.molstruc.2021.132134.

[ref9] Vaughn A. E.; Deshmukh M. Glucose Metabolism Inhibits Apoptosis in Neurons and Cancer Cells by Redox Inactivation of Cytochrome c. Nat. Cell Biol. 2008, 10 (12), 1477 10.1038/ncb1807. 19029908 PMC2626347

[ref10] Abramczyk H.; Brozek-Pluska B.; Kopec M.; Surmacki J.; Błaszczyk M.; Radek M. Redox Imbalance and Biochemical Changes in Cancer by Probing Redox-Sensitive Mitochondrial Cytochromes in Label-Free Visible Resonance Raman Imaging. Cancers 2021, 13 (5), 960 10.3390/cancers13050960. 33668874 PMC7956250

[ref11] Eleftheriadis T.; Pissas G.; Liakopoulos V.; Stefanidis I. Cytochrome c as a Potentially Clinical Useful Marker of Mitochondrial and Cellular Damage. Front. Immunol. 2016, 7, 279 10.3389/fimmu.2016.00279. 27489552 PMC4951490

[ref12] Roh J. S.; Sohn D. H. Damage-Associated Molecular Patterns in Inflammatory Diseases. Immune Network 2018, 18 (4), e27 10.4110/in.2018.18.e27. 30181915 PMC6117512

[ref13] Falschlehner C.; Schaefer U.; Walczak H. Following TRAIL’s Path in the Immune System. Immunology 2009, 127 (2), 145–154. 10.1111/j.1365-2567.2009.03058.x. 19476510 PMC2691779

[ref14] Seder R. A.; Paul W. E. Acquisition of Lymphokine-Producing Phenotype by CD4+ T Cells. Annu. Rev. Immunol. 1994, 12, 635–673. 10.1146/annurev.iy.12.040194.003223. 7912089

[ref15] Romagnani S. Lymphokine Production by Human T Cells in Disease States. Annu. Rev. Immunol. 1994, 12, 227–257. 10.1146/annurev.iy.12.040194.001303. 8011282

[ref16] Unsay J. D.; Cosentino K.; Subburaj Y.; García-Sáez A. J. Cardiolipin Effects on Membrane Structure and Dynamics. Langmuir 2013, 29 (51), 15878–15887. 10.1021/la402669z. 23962277

[ref17] Yin V.; Shaw G. S.; Konermann L. Cytochrome c as a Peroxidase: Activation of the Precatalytic Native State by H2O2-Induced Covalent Modifications. J. Am. Chem. Soc. 2017, 139 (44), 15701–15709. 10.1021/jacs.7b07106. 29048162

[ref18] Li M.; Sun W.; Tyurin V. A.; DeLucia M.; Ahn J.; Kagan V. E.; Van Der Wel P. C. A. Activation of Cytochrome C Peroxidase Function Through Coordinated Foldon Loop Dynamics upon Interaction with Anionic Lipids. J. Mol. Biol. 2021, 433 (15), 167057 10.1016/j.jmb.2021.167057. 34033821 PMC8380053

[ref19] Basova L. V.; Kurnikov I. V.; Wang L.; Ritov V. B.; Belikova N. A.; Vlasova I. I.; Pacheco A. A.; Winnica D. E.; Peterson J.; Bayir H.; Waldeck D. H.; Kagan V. E. Cardiolipin Switch in Mitochondria: Shutting off the Reduction of Cytochrome c and Turning on the Peroxidase Activity. Biochemistry 2007, 46 (11), 3423–3434. 10.1021/bi061854k. 17319652 PMC3356783

[ref20] Fiorucci L.; Erba F.; Santucci R.; Sinibaldi F. Cytochrome cInteraction with Cardiolipin Plays a Key Role in Cell Apoptosis: Implications for Human Diseases. Symmetry 2022, 14 (4), 767 10.3390/sym14040767.

[ref21] Moody P. C. E.; Raven E. L. The Nature and Reactivity of Ferryl Heme in Compounds I and II. Acc. Chem. Res. 2018, 51 (2), 427–435. 10.1021/acs.accounts.7b00463. 29327921

[ref22] Bettini E.; Locci M. SARS-CoV-2 MRNA Vaccines: Immunological Mechanism and Beyond. Vaccines 2021, 9 (2), 147 10.3390/vaccines9020147. 33673048 PMC7918810

